# Which Role for Muscle-Sparing Posterolateral Thoracotomy in the Treatment of Spontaneous Pneumothorax?

**DOI:** 10.1055/s-0043-1770954

**Published:** 2023-12-28

**Authors:** Ibrahim Issoufou, Rabiou Sani, Daouda Amadou, Kadre Alio, Kaled Adamou-Nouhou, Marouane Lakranbi, Rachid Sani, Yassine Ouadnouni, Habibou Abarchi, Mohamed Smahi

**Affiliations:** 1 Department of Thoracic Surgery, Teaching Hospital Hassan II, Fez, Morocco; 2 Department of Cardiovascular Surgery, Teaching Hospital Hassan II, Fez, Morocco; 3 Department of Stomatology and Maxillofacial Surgery, Hôpital Général de Référence, Niamey, Niger; 4 Department of Surgery and Surgical Specialities, Faculty of Health Sciences, Abdou Moumouni University, Niamey, Niger; 5 Department of Surgery, Faculty of Medicine and Pharmacy, University Sidi Mohamed Ben Adellah, Fez, Morocco; 6 Department of General Surgery, National Hospital of Niamey, Niamey, Niger; 7 Department of Pediatric Surgery, Hospital Amirou Boubacar Diallo, Niamey, Niger

**Keywords:** pneumothorax, spontaneous pneumothorax, thoracocentesis, pleural tuberculosis, thoracotomy

## Abstract

**Objective** This study aims to show the place of muscle-sparing posterolateral thoracotomy in the treatment of spontaneous pneumothorax.

**Methods** It was a single-center study performed in the Department of Thoracic Surgery of Teaching hospital Hassan II of Fez for 8 years. We adopted the nosological definition, which classifies spontaneous pneumothorax into three categories. We included patients over 15 years of age with primary or secondary spontaneous pneumothorax operated by posterolateral thoracotomy without muscle section, and we analyzed the specific indications of this approach. It included 49 patients with primary or secondary spontaneous pneumothorax, operated by muscle-sparing posterolateral thoracotomy. Data were collected from regularly updated computer files of patients, entered by Excel 2013, and analyzed using SPSS.20 software. These data are: epidemiological, clinical, radiological, surgical exploration, surgical procedure, the result of the surgery and the evolution.

**Results** The average age was 42 years. Smoking was found in 61% of cases and pulmonary tuberculosis in 10% of cases. Thoracic computed tomography (CT) showed bullae and blebs in 31% of cases, pleural adhesions and pachypleuritis in 50% of cases, and hydropneumothorax with pachypleuritis in 37% of cases. There is a statistical correlation between pleuropulmonary decortication and pachypleuritis ( *p* = 0.002) or hydropneumothorax ( *p* = 0.001) on CT. Bullae and blebs resection was performed in 53% of cases and pleuropulmonary decortication in 63% of cases. A right pleuropneumonectomy was performed in one case. The follow-up was uneventful in 82% of cases.

**Conclusion** Muscle-sparing posterolateral thoracotomy remains the best approach and leads to good results.

Surgery remains the cornerstone for managing of recurrent spontaneous pneumothorax. In the era of minimally invasive approaches, this surgery remains largely dominated by video-assisted thoracoscopic surgery (VATS) and thoracoscopy. Developed in the earliest of this century, these minimally invasive approaches have supplanted open surgery because of their many advantages. The latter tends to be shelved except in some clinical circumstances, which remain for the majority linked to the difficulties of developing countries. This study intends to show the value of posterolateral thoracotomy without muscle section in the treatment of spontaneous pneumothorax and to compare its indications with the minimally invasive approaches.

## Methods

**Fig. 1:**
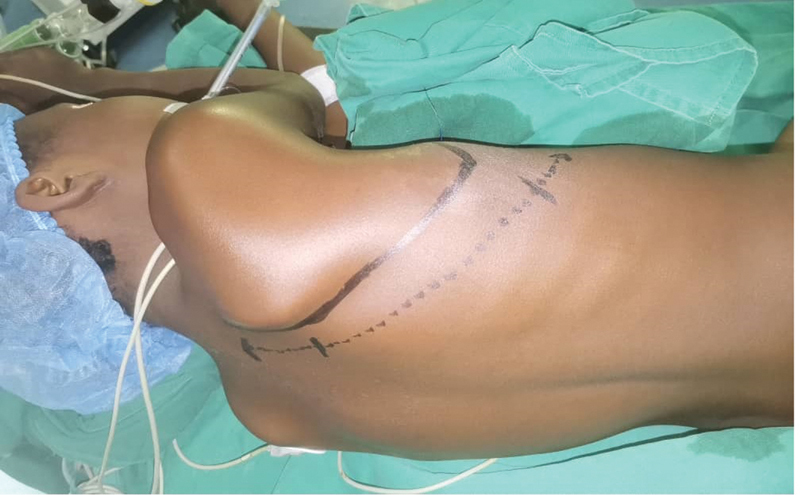
Line of incision (dotted).

**Fig. 2:**
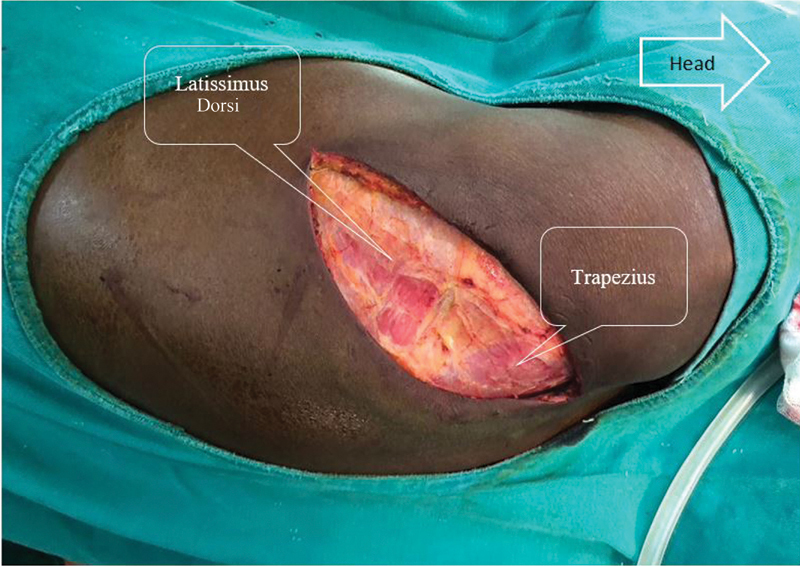
The superficial plane of the muscles during thoracotomy.

**Fig. 3:**
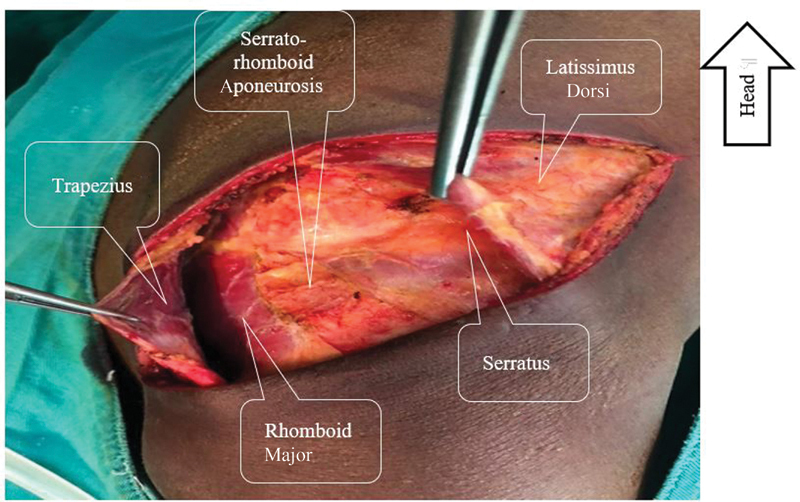
The deep muscular plane represented by the rhomboid major and the serratus connected by the serratorhomboid aponeurosis. All these muscles must not be sectioned but separated.

**Fig. 4:**
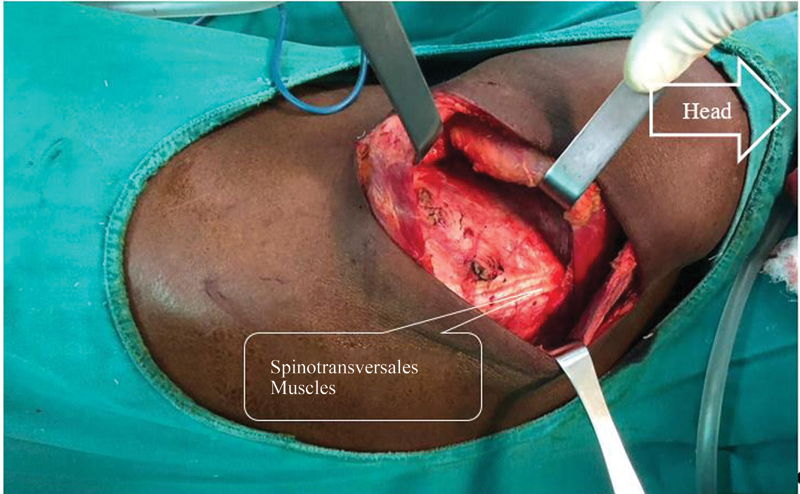
After opening the aponeurosis without any section of the muscles, we find the intercostal plane with the spinotransversales muscles.

**Fig. 5:**
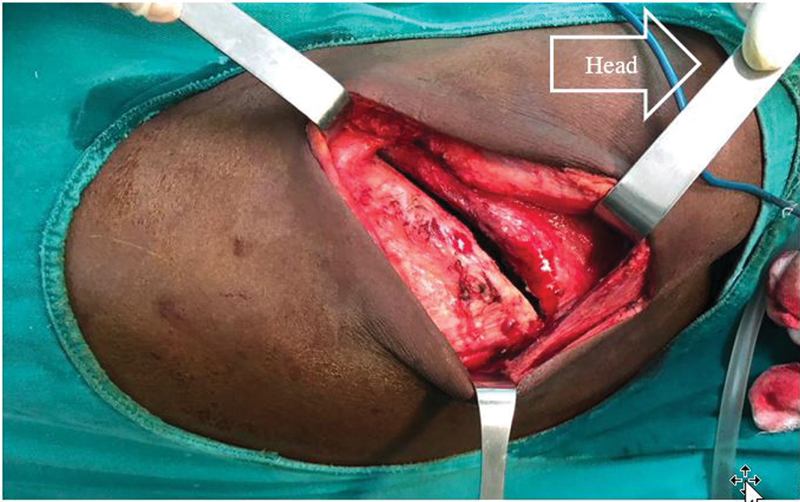
The thoracotomy is performed by incising the upper edge of the selected rib.

We adopted the nosological definition that classifies spontaneous pneumothorax into three categories: primary or idiopathic spontaneous pneumothorax, secondary spontaneous pneumothorax that occurs on pathological lung, and catamenial pneumothorax occurring in women premenopausal. This is a single-center, descriptive, and analytical study, performed in the Thoracic Surgery Department of Teaching hospital Hassan II in Fez over a period of 8 years (2010–2017). We included patients over 15 years of age with primary or secondary spontaneous pneumothorax operated by posterolateral thoracotomy without muscle section, and we analyzed the specific indications of this approach. This posterolateral thoracotomy is performed in the lateral decubitus position, the patient is lying on the healthy side, with a block placed under the scapula. The upper arm on the side of the thoracotomy is extended in front of the head. Care is taken to ensure that the tracheal intubation tube is maintained properly in place. This method hides the scapula thus giving better exposure of the operating field. The line of the incision makes a bisector from the angle formed by the line of the spinous processes and a line passing through the spinal edge of the scapula ( Fig. 1 ). After a cutaneous detachment, a dissection of the superficial muscle plane without sectioning the muscles is performed. This muscle plane includes the latissimus dorsi muscle in front and the trapezius muscle behind ( Fig. 2 ). Their spacing with the help of two Farabeuf reveals the deep muscular plane, which comprises the serratus anterior muscle (or serratus anterior) in front and the rhomboid muscle behind ( Fig. 3 ). The aponeurosis uniting these two muscles, known as the serratorhomboid aponeurosis, is incised and opened from front to back without cutting the two muscles. Thus, the costal plane is exposed and the choice of the intercostal space for thoracotomy is made easy ( Fig. 4 ). The thoracotomy is performed by incising the upper edge of the selected rib ( Fig. 5 ) and then we proceed to the intercostal spacing using a retractor.

Regarding the indications for surgical treatment in general, we selected patients with ongoing air leak or nonresolving pneumothoraxes on chest X-ray beyond 5 days as well as the first homo or contralateral recurrences. No case of catamenial pneumothorax has been recorded. In patients with a first episode, the initial management (including chest drainage) is performed by the pulmonologist who refers the patient for surgery when the outcome was unfavorable. Data were collected from regularly updated computer files of patients, entered by Excel 2013, and analyzed using SPSS.20 software. These are epidemiological, clinical, and radiological data, surgical exploration, surgical procedure, the result of the surgery and the evolution.

## Results

Our study covered 49 spontaneous pneumothoraxes operated by posterolateral thoracotomy without muscle section. The average age of the patients was 42 ± 14 years, men being in the majority with 94% of cases (46 men for 3 women). Smoking is found in 61% of cases ( *n* = 30). The other antecedents were pulmonary tuberculosis in 10% of patients ( *n* = 5), including one patient treated three times, Marfan's disease, asthma, and arterial hypertension in one case treated once. One patient was operated on for osteosarcoma of the knee and received chemotherapy. His radiological assessment did not show any metastatic lung lesion, nor bullae or blebs. Clinically, the association with dyspnea and chest pain was predominant in 96% of cases ( *n* = 47). Most of these patients present a fever was found, purulent sputum with hemoptysis in one case and dyspnea in another case. The average time between the onset of these symptoms and the surgical consultation was 35 ± 26 days. The chest X-ray showed pneumothorax on the right side in 55% of cases, on the left side in 43% of cases and bilateral side in one case. This pneumothorax was larger in 63% of cases ( *n* = 31), with a liquid level in 27% of cases ( *n* = 13) and loculated in 10% of cases ( *n* = 5). The chest computed tomography (CT) showed bullae and blebs in 31% of cases ( *n* = 15), an emphysematous lung in 51% of cases ( *n* = 25), pachypleuritis in 50% of cases, a hydropneumothorax with pachypleuritis in 37% of cases, and hemopneumothorax in 8% of cases ( *n* = 4). There is a statistical correlation between pleuropulmonary decortication and the presence on CT of pachypleuritis ( *p* = 0.002) or hydropneumothorax ( *p* = 0.001). The other lesions were tuberculosis destroyed lung in one case, a ruptured hydratid lung cyst in the pleura in one case, a tuberculosis and malaria in one case, a bilateral lung fibrosis in one case, and bronchiectasis in another case. Chest tube was inserted in 84% of patients ( *n* = 41) and exsufflation in two patients. The mean duration of chest tube was 22 days (extreme: 3–120 d). Seventy-one percent of patients ( *n* = 35) kept their chest tube more than 5 days (average: 23 ± 20 d) due to persistent air leak and/or pachypleuritis. Pneumothorax was classified as primary in 22% of patients ( *n* = 11) and secondary in 78% of cases ( *n* = 38). A first-time pneumothorax was found in 76% of the cases ( *n* = 37), whereas in 24% of the cases ( *n* = 6) it was a recurrence. Thus, the etiologies ( Table 1 ) in secondary pneumothorax were chronic obstructive pulmonary disease (COPD) in 55% of cases ( *n* = 27), active tuberculosis in 12% of cases ( *n* = 6) and in every one case, a destroyed lung after tuberculosis, asthma, Marfan's disease, bronchiectasis sequels of tuberculosis, and a ruptured hydratid cyst in the pleura. Regarding surgical approach, pachypleuritis was detached through the extrapleural plane in 60% of patients ( *n* = 29) because of its thickness. Intraoperative exploration found apical bullae and blebs in 53% of cases ( *n* = 26) and in one case they were located on the left lower lobe, which was destroyed. The intraoperative exploration also found upper lobes encrusted in the mediastinum and lower ones against the diaphragm in 67% of cases ( *n* = 33), pus in 18% of cases ( *n* = 9), hemothorax with clots in 10% of cases ( *n* = 5), and in each case loculated pleural effusion, a ruptured hydratid cyst in the pleura cavity with bronchial fistula, a destroyed lung by tuberculosis, and a middle and a lower right lobe destroyed by tuberculosis. The surgical approach ( Table 2 ) was bullectomy and blebs resection in 53% of cases (with linear stapling in 77%), pleuropulmonary decortication in 63% of cases ( *n* = 31), and pulmonary peeling in 14% of cases ( *n* = 7), removing of pleural clots in 10% of cases ( *n* = 5), and pleurodesis in 37% of cases ( *n* = 18). The lung resections were in each case, a right pleuropneumonectomy, a lower bilobectomy, a wedge resection, and a percystectomy with suture of bronchial fistulas. The postoperative result was uneventful in 82% of cases ( *n* = 41). Eighteen percent of the patients ( *n* = 9) stayed for 1.80 ± 7 days in intensive care unit. The morbidity rate was 16%, whereas there is no mortality. These complications ( Table 3 ) were a wall infection in three cases, a pneumonia in two cases, a septic shock in one case, and a hemothorax requiring a thoracotomy in one case. One patient presented a postoperative empyema after lower bilobectomy requiring open-window thoracostomy with a good outcome due to spontaneous closure of the thoracostomy after 1 year. The average hospitalization duration was 9 ± 6 days. The histological examination revealed an active tuberculosis in 8% of cases ( *n* = 4; including the case of empyema after lower bilobectomy). Three of these patients were already known with tuberculosis under treatment and the fourth one also started his treatment with an overall good outcome. Except for four patients who stopped their follow-up, the outcome was uneventful for all others (92% of cases) after a mean follow-up of 36 ± 26 months without any recurrence of the pneumothorax.

**Table 1:** Etiologies of secondary pneumothorax

Etiologies	Percentage
Chronic obstructive pulmonary disease	55
Active tuberculosis	12
Destroyed lung after tuberculosis	2 (1 case)
Asthma	2 (1 case)
Marfan's disease	2 (1 case)
Bronchiectasis sequels of tuberculosis	2 (1 case)
Ruptured hydratid cyst in the pleura	2 (1 case)

**Table 2:** Surgical approaches

Surgical approaches	Percentage
Bullectomy and blebs resection	53
Pleuropulmonary decortication	63
Pulmonary peeling	14
Removing of pleural clots	10
Pleurodesis	37
Pleuroneumonectomy	2 (1 case)
Lower bilobectomy	2 (1 case)
Wedge resection	2 (1 case)
Percystectomy with suture of bronchial fistulas	2 (1 case)

**Table 3:** Postoperative complications

Complications	Percentage
Wall infection	6
Pneumonia	4
Septic shock	2 (1 case)
Hemothorax requiring a thoracotomy	2 (1 case)
Empyema requiring open-window thoracostomy	2 (1 case)

## Discussion

Spontaneous pneumothoraxes are benign diseases that are widespread all over the world. The incidence is estimated at 16.7/100,000 per year for men and 5.8/100,000 per year for women. 1 The male predominance is reported by several studies with female/male ratios varying from 1:2 to 1:6. 2 In our study, this ratio was 1/15. This is because of the prevalence of smoking in males, which is an important risk factor for the occurrence of these pneumothoraxes and their recurrence. Indeed, 61% of the patients in our study were smokers, and all were men. In a British study 3 nearly 80% of secondary pneumothoraxes were associated to smoking and COPD, whereas in our study it is only 55%. These etiologies vary according to the diseases that the population is exposed to. In our Moroccan context and in many developing countries, beyond the lung diseases caused by smoking, infectious diseases in particular tuberculosis and pulmonary hydatidosis are also frequent. This epidemiological peculiarity would explain the preponderance of secondary spontaneous pneumothorax in our study (78%), whereas the primary forms were more frequent in certain western series (85%). 4 Thus, a tuberculosis origin (sequels or active lesions) was found in 26.5% of secondary pneumothoraxes in our series. The lesions were mainly a destroyed lung and bronchiectasis following tuberculosis or miliary tuberculosis. Emad et al in an Egyptian study also found tuberculosis in almost 18% of cases. 5 In one case, the rupture of a hydratid cyst was the cause of the pneumothorax. This etiology is rare and is only found in countries where hydatidosis is endemic. Its frequency varies from 2.4 to 6.2% among secondary pneumothoraxes. 6 These latter occur when the ruptured cyst in the pleura connects a bronchus fistula with the pleural cavity.

The indications for surgery in spontaneous pneumothorax are well known. 7 In our patients, these indications were due to recurrence of pneumothorax and persistent air leak since 71% of patients have their chest tube more than 5 days. On the other hand, the choice of the surgical approach remains a subject of discussion between the authors, each with its advantages and disadvantages. 1 In the case of our patients, we performed the posterolateral thoracotomy because of the following:

- The need for pleuropulmonary decortication to release the lung from the pachypleuritis. This pachypleuritis is seen on the CT scan in most patients and also found during surgical exploration. The thickness of the latter imposed the approach of the pleural cavity through an extrapleural plane. VATS surgery could be possible in the early phases of the empyema, especially before the formation of a thick pachypleuritis. 8 9 But when this pachypleuritis is wide, sometimes exceeding 2 cm, open surgery is recommended. 10 11
- The chronicity of pneumothorax that promotes infection of the pleural cavity and the development of fibrous adhesions within the diaphragm and mediastinum such as those found in 67% of our patients. This chronicity is due to excessive duration of chest tube drainage before surgery and the quality of the underlying lung. For example, Malhotra et al found a longer duration of drainage in the event of associated tuberculosis. 12 In these situations, the risk of conversion after VATS increases from 22 to 86% when the duration of symptoms' evolution/duration of symptoms is greater than 16 days. 13
- The destroyed lung by tuberculosis, which is the consequence of poorly treated or untreated or recurrent tuberculosis, as for our patient who was treated three times. 14 The chronic inflammatory and infectious process lead to the destruction of the lung and the development of pachypleuritis with pleural symphysis sometimes requiring pleuropneumonectomy like the case of our patient. 15 This laborious and hemorrhagic surgery sometimes requires costal resection during thoracotomy because of the very tight symphysis. 16 To date, no case of extrapleural pneumonectomy by VATS has been reported in the literature for posttuberculous destroyed lung.
- The pleural ruptured hydatid cyst with bronchial fistulas. It complicates hydatid cysts of the lung, which are large, peripheral, or infected. 17 Thoracoscopic surgery cannot be considered in these cases because of the pachypleuritis requiring a decortication, the closing of the bronchial fistulas, and the treatment of the residual cavity of the cyst. 6 Admittedly, certain types of hydatid cysts can be operated on by VATS. 18 19 But in cases of complicated hydatid cyst, such as the case of our patient who presented with pachypleuritis and a bronchopleural fistula, a thoracotomy approach remains preferable. 10 11

Thus, the importance of pleural and lung lesions associated with pneumothorax explains the plurality of surgical approach performed in our patients apart from the resections of classic bullae and blebs found in most surgical series. 20 21 Despite the complexity of these surgical procedures and the preponderance of secondary pneumothoraxes causing recurrence, the results remain satisfactory since no recurrence of pneumothorax was recorded during the follow-up period. This is due to on one side to the surgical approach itself since recurrences are less frequent with thoracotomy compared with video-assisted surgery (1 vs. 5%) 4 and on the other side to the decortication allowing a tighter pleural symphysis preventing any recurrence of the pneumothorax. The observed morbidity of 18% was mainly due to septic complications. These kinds of complications are unusual in pneumothorax surgery. 20 22 They are related to the secondary nature of these pneumothoraxes, 4 their chronicity, and the underlying tuberculosis.

## Conclusion

Spontaneous pneumothoraxes are diseases for which the management is simple and well known in western countries. Their etiologies may vary depending on the geographic context. Delay in treatment can lead to sometimes extremely serious complications that the thoracic surgeon must adapt to provide effective treatment. The posterolateral thoracotomy without muscle section remains for us the best approach in this management and leads to good results.
